# Long-Term Impact of Multiple Micronutrient Supplementation on Micronutrient Status, Hemoglobin Level, and Growth in Children 24 to 59 Months of Age: A Non-Randomized Community-Based Trial from Pakistan

**DOI:** 10.3390/nu15071690

**Published:** 2023-03-30

**Authors:** Aslam Khan, Zia Ul-Haq, Sadia Fatima, Jawad Ahmed, Hussah M. Alobaid, Sheraz Fazid, Nawshad Muhammad, Cecilia Garzon, Yasir Ihtesham, Ijaz Habib, Mahamadou Tanimoune, Khalid Iqbal, Muhammad Arshad, Sher Zaman Safi

**Affiliations:** 1 Institute of Basic Medical Science, Khyber Medical University, Peshawar 25120, Pakistan; 2 Institute of Public Health Sciences, Khyber Medical University, Peshawar 25120, Pakistan; 3 Institute of Health & Wellbeing, University of Glasgow, Glasgow G12 8QQ, UK; 4 Department of Zoology, College of Science, King Saud University, Riyadh 11362, Saudi Arabia; 5 World Food Programme, Islamabad 44000, Pakistan; 6 World Food Programme, Peshawar 25000, Pakistan; 7 Jhang Campus, University of Veterinary and Animal Sciences, Lahore 54000, Pakistan; 8 Faculty of Medicine, Bioscience and Nursing, MAHSA University, Jenjarom 42610, Malaysia; 9 Interdisciplinary Research Center in Biomedical Materials, COMSATS University Islamabad Lahore Campus, Lahore 54000, Pakistan

**Keywords:** stunting, malnutrition, MNP, vitamin A, vitamin D, zinc, growth parameter, anemia

## Abstract

Cost-effective interventions are needed to address undernutrition, particularly micronutrient deficiencies, which are common in children under the age of five in low- and middle-income countries. A community-based, non-randomized clinical trial was undertaken in the Kurram district of Khyber Pakhtunkhwa from January 2018 to June 2019, to evaluate the effect of locally produced micronutrient powder (local name: Vita-Mixe) on plasma micronutrient status, hemoglobin level, and anthropometric outcomes. Children aged 24–48 months old were recruited and allocated to the intervention and control arm of the study. The enrolled children in the intervention arm received one micronutrient powder (MNP) sachet for consumption on alternate days for 12 months. To assess the impact of the intervention on plasma levels of zinc, vitamin D, vitamin A, and hemoglobin level, blood samples were taken at baseline and after one year following the intervention. The analysis was conducted using Enzyme-Linked Immunosorbent Assay (ELISA), atomic absorption spectrometry, and an automated hematology analyzer. For the impact on growth parameters, the anthropometric assessment was performed using WHO standard guidelines. A 24 h dietary recall interview was used to assess the nutrient intake adequacy. Results showed that in the intervention arm, children had on average a 7.52 ng/mL (95% CI 5.11–9.92, *p*-value < 0.001) increase in the plasma level of vitamin A, 4.80 ng/mL (95% CI 1.63–7.95, *p*-value < 0.002) increase in vitamin D levels and 33.85 µg/dL (95% CI 24.40–43.30, *p*-value < 0.001) increase in the plasma zinc level, as well as a 2.0g/dL (95% CI 1.64–2.40, *p*-value < 0.001) increase in hemoglobin level. Statistically significant improvement was observed in the weight-for-height z-score (WHZ) (from −1.0 ± 0.88 to −0.40 ± 1.01, *p* < 0.001) and weight-for-age z-score (WAZ) (from −1.40 ± 0.50 to −1.05 ± 0.49, *p* < 0.001) in the intervention group compared to the control group. No statistically significant change was observed in the height-for-age z-score (HAZ) in the intervention group (*p* = 0.93). In conclusion, micronutrient powder supplementation is a cost-effective intervention to improve the micronutrient status, hemoglobin level, and growth parameters in under-five children, which can be scaled up in the existing health system to address the alarming rates of undernutrition in Pakistan and other developing countries.

## 1. Introduction

Undernutrition, especially micronutrient malnutrition, in pre-school children is a major concern of public health in low- and middle-income countries [1]. Annually, undernutrition causes about 13.5 million deaths in children under the age of five [2]. In 2020, an estimated 47 million children under the age of five years were wasted, among which 14.3 million are severely wasted and 144 million children were stunted [3]. Recent estimates show that the global burden of micronutrient insufficiency is 56% (372 million) in pre-school children and 69% (1.2 billion) in women of reproductive age. Regionally, 99 million children (aged 6–59 months) with micronutrient deficiency live in South Asia, 98 million live in Sub-Saharan Africa, and 85 million in East Asia and the Pacific [4].

According to the National Nutrition Survey of Pakistan (NNS-2018), the burden of undernutrition among 6–59-month-old children is very high, i.e., stunting 40%, wasting 17.7%, and underweight 28.9%. Similarly, the prevalence of micronutrient deficiencies among these children is also very high, i.e., vitamin A 51.5%, vitamin D 62.7%, and zinc 18.6%. Moreover, iron deficiency anemia was estimated at 28.6%. The Khyber Pakhtunkhwa Newly Merged District (KP-NMD) presents the worst situation of undernutrition in the country. Micronutrient deficiencies are above the national average, i.e., vitamin A 54.9% and vitamin D 81.5%. Only zinc deficiency is comparatively less than the national average, i.e., 16.6% [5]. Such circumstances affect the most vulnerable parts of the population, especially children under the age of five, as well as pregnant and lactating women, with malnutrition appearing as one of the major concerns.

Children and pregnant women require minerals and vitamins for growth and normal physiological function, which are frequently unmet by dietary intake [6,7]. These deficiencies in children may lead to anemia [8], growth retardation [9], neurological disorders [10], and poor immunity [11]. In general, undernutrition negatively affects a population’s health and productivity over the long run.

Micronutrient deficiencies also contribute to other health problems, such as iron deficiency anemia and intellectual impairments [12]. Similarly, vitamin A deficiency can potentially impair vision and is linked to higher rates of morbidity and mortality in children [13]. Vitamin D insufficiency causes poor bone formation and mineralization, which causes rickets in children and osteoporosis in adults [14]. Likewise, zinc deficiency affects growth and compromises immunity leading to diarrhea and respiratory infections [15].

In light of the current prevalence and consequences of micronutrient deficiencies, the provision of micronutrient supplementation to children under the age of five is critical for reducing the burden of micronutrient deficiencies. Multiple micronutrient supplementation with vitamins and minerals is found to be a cost-effective strategy for reducing micronutrient deficiencies in children [16]. MNP supplementations have been shown to improve micronutrient status, hemoglobin levels, and other growth parameters in pre-school children [17,18,19]. Though the findings of the already conducted studies are promising with regards enhancing the micronutrient level of children, further evidence is required to evaluate the impact of MNP supplementation at the community level, particularly among those with limited resources and weak health service infrastructure. Further, evidence on the impact of MNPs on plasma micronutrient levels (zinc, vitamin A, and vitamin D) and their relationship with anemia status in children aged between 24 and 59 months at the community level, particularly in a resource-limited setting such as the Pakistani context, is scarce. Studies that have already been carried out in Pakistan have solely considered the impact of MNPs on children’s anthropometric outcomes and anemia status. By contrast, the purpose of this intervention research was to explore the effect of MNPs on micronutrient status, hemoglobin level, and growth parameters in 24–59-month-old children in one of the most deprived areas of Pakistan. In addition, the impact of MNPs on vitamin D levels has been investigated, which is rarely reported in the literature.

## 2. Methodology

### 2.1. Study Design and Participants

This study is a part of the project “Ready to Use Supplementary Foods (RUSF) to Prevent Stunting Among Children Under Five Years in Kurram Agency”, a community-based non-randomized cluster-controlled clinical trial, undertaken from January 2018 to December 2020 in the low-resource rural areas of the tribal district Kurram of Khyber Pakhtunkhwa, Pakistan.

In Pakistan, community health services at household levels are provided through the Lady Health Workers Program. One Lady Health Worker (LHW) is responsible for 100–150 households in her covered (assigned) area. In the current study, each LHW-covered area was considered an independent cluster. Furthermore, the clusters were refined using the expanded program on immunization (EPI) maps. Intervention and control clusters were selected based on natural segregation, especially prominent demarcation, to avoid contamination of the intervention between the study groups.

A total of 80 matched clusters were included in the main trial, 40 each in the intervention and control arms. The overall sample size of the project was 7200 study participants from all four groups (pregnant and lactating women, 6–23 months, and 24–59 months children). Among the total participants, there were 1840 children between the ages of 24 and 59 months. In the current study, the impact of MNP supplementation was investigated in a sub-sample of 24–59-month-old-children. The trial is registered with ISRCTN 94319790.

In this study, the MNP effect on plasma micronutrient level was evaluated in those households that had at least one child between 24 and 48 months old at the time of enrollment in both the intervention and control arms. Households with children aged 24–48 months were identified using the expanded program on routine immunization (EPI) maps in the respective areas. First, a line listing of the households in the clusters was performed and then systematic random sampling was used to select households containing at least one child aged 24–48 months. In the third step, one eligible participant from each household was included in the trial.

### 2.2. Sample Size Estimation

A 2-by-2 repeated measures design was adopted, with two groups of participants being measured at two different time points. The primary goal of the study was to compare the change in the intervention group to the change in the control group over time. Sample sizes were calculated for all study variables, including vitamin A, vitamin D, zinc, and hemoglobin. The maximum sample size was determined for hemoglobin; thus, the sample size estimation for this investigation was based on hemoglobin levels. A sample size of 47 in the intervention group and 47 in the control group achieved 81% power to detect a difference in mean changes of 0.61 g/dL with an SD of 1 at the first time point, an SD of 0.88 at the second time point, and a correlation of 0.4 between measurement pairs. With a 20% attrition rate, the sample size was extended to 58 in each group. The significance level (alpha) for a two-sided, two-sample t-test was set at 0.05 [20]. As a result, 111 children aged 24–59 months (58 in the intervention arm and 53 in the control arm) were chosen and participated in the trial after their parents/guardians gave informed consent.

### 2.3. Inclusion and Exclusion Criteria

**Figure 1:**
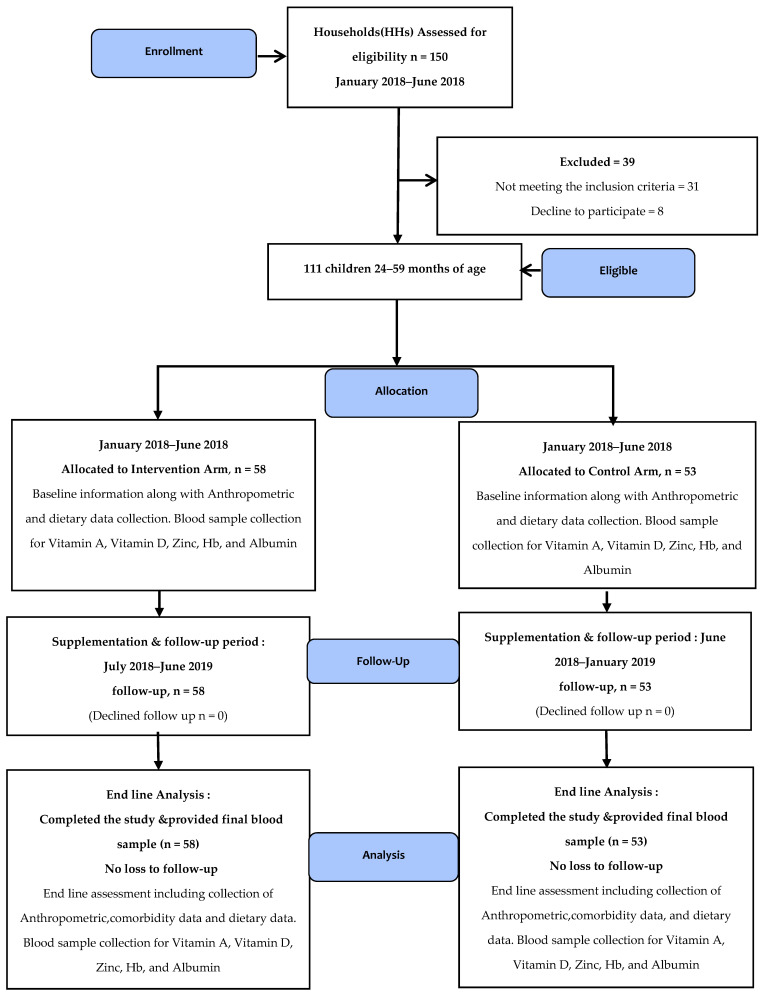
Consort diagram.

All children (24–48 months) at the time of enrollment were included in the trial. However, children with severe malnutrition, i.e., Mid Upper Arm Circumference < 11.5 cm (MUAC), congenital malformations identified at the baseline or severe developmental impairments such as cerebral palsy, those with malabsorption/metabolic disorders or malignancy, as well as those children who were allergic to the supplement ingredients, had persistent diarrhea, or who were unable to take MNP (e.g., cleft palate), and children who had been regularly taking other supplements or had any known gastrointestinal tract disorders, were excluded from the study (Figure 1).

### 2.4. MNP Intervention, Composition, Dosage, Distribution, and Utilization

Micronutrient Powder (MNP) for Children (local name: Vita-Mixe, Ready-to-Use Supplementary Foods) was provided to children aged 24–59 months. The composition per one-gram sachet of MNP (homogeneous, dried, and stable) is given in Table 1. One MNP sachet (1 g) on each alternate day was provided to the child for a period of 12 months, from July 2018 to June 2019. For a whole year, the enrolled child received a total of 15 MNP sachets per month which provided an additional intake of 50% RNI/day for each micronutrient, which is justifiable in the majority of circumstances for children aged under 5 years. Most micronutrients were delivered under the recommended dietary allowance [21]. The preparation of micronutrient powder complied with international standards, best practices, and/or recommendations for quality and food safety management. This MNP ration was provided by the UN World Food Program. These supplements were delivered by Lady Health Workers (LHW) on a monthly basis as a food package from the near healthcare facility for the recruited study participants in their households. There were no adverse events reported in the overall study. Lady Health Workers checked the utilization of the MNP on regular visits to all the households of the study participants to record the supplement use, and they collected empty sachets to monitor compliance. It was ensured that the supplement was given to the targeted child on an alternate day and they encouraged continued use of the supplement.

### 2.5. Data Collection

At the baseline, demographic, socioeconomic characteristics of the mothers/caregivers, dietary recall data, comorbidity, Infant and Young Child Feeding practices (IYCF), vaccination status, anthropometric measurements, and blood samples of children were collected as per the standard protocol. The child’s weight and height were measured using digital weighing scales (SECA) and height boards (SECA). Weight was measured in light clothing to the nearest 100 g, while height was measured without shoes and caps to the nearest 0.1 cm. As for the MUAC, the measurement was taken to the nearest 1 mm (flexible UNICEF insertion tape) as per the WHO standard protocol [22]. Dietary intake was analyzed by trained field staff using a multiple-pass 24 h dietary recall questionnaire at two time points, i.e., at the baseline and at the end line of the study [23]. The aim was to determine the typical nutritional intake in the community only. The follow-up data were collected at the community level by a trained data collection team with the assistance of Lady Health Workers in their respective areas. At the end line, anthropometric data, comorbid conditions of the child (diarrhea and acute respiratory diseases) in the last month, and blood samples were collected from each participant. No loss to follow-up was reported among the participants recruited for the biochemical sampling and the same participants were observed at the end line of the trial.

### 2.6. Data Quality Control and Quality Assurance

To validate the study data, a verification team (not the data collectors) randomly visited a few of the households to double-check the data accuracy. A supervisor was assigned to each cluster to keep an eye on the data enumerators and collectors. The entire data collection process was quarterly monitored by the project manager, PI, and co-PI.

### 2.7. Biochemical Analysis

Blood samples (non-fasting) were collected from each participant with venipuncture by a skilled phlebotomist at the basic health unit. For plasma zinc assessment, the blood was collected in trace element-free tubes to avoid contamination. All samples were centrifuged to obtain plasma/serum in the pathology laboratory of DHQ hospital Parachinar Newly Merged District of Khyber Paktunkhawa (NMD-KPK) Pakistan. The separated plasma/serum was tightly packed in Eppendorf tubes and was transported in dry ice on a daily basis to the Biochemistry laboratory of Khyber Medical University for processing and storage at −80 °C until further biochemical analysis. The plasma/serum was used for the measurement of plasma albumin using a fully automated chemistry analyzer (Cobas 6000) (Roche Diagnostics, Switzerland, Rotkreuz). The 25-hydroxy vitamin D was analyzed with the ELISA technique (according to its available literature protocol) using BIOS Microwell Diagnostic System (Perkin Elmer, catalog No: 10501, USA) with a cut-off value of <30 ng/mL. The vitamin A (retinol) was analyzed through an ELISA kit (Elabscience, catalog No: E-EL-0135, Wuhan, China) with a cut-off value of <18.8 ng/mL. To measure the plasma zinc level, a proper wet acid digestion method was used to digest plasma and then followed by analysis through an atomic absorption spectrometer (model: AAS 700, make: Perkin Elmer, Waltham, MA, USA) with a cut-off value of <65 µg/dL [24]. The whole blood samples were used to determine the hemoglobin level of the participant using an automated hematology analyzer (Medonic M-Series Hematology Analyzer, Sweden) with a cut-off value of <11.00 g/dL. These assays were carried out following the instructions of the manufacturer. Laboratory quality control assessment was measured with the Westgard rule on the Levey–Jennings chart [25].

### 2.8. Statistical Analyses

STATA version 15 was used to analyze the data. The distribution of the data was checked for skewness and kurtosis for each of the micronutrient variables, followed by a graphical cross-verification by making a histogram plot against the normal distribution curve; the data were found to be normally distributed. A Chi-square test was used to evaluate the difference between child characteristics and the study group at the baseline for categorical variables, whereas a t-test was used for continuous variables. A paired sample *t*-test was used to assess the association between the use of MNPs and plasma micronutrients (between group mean differences at baseline and end line), child anthropometric assessment, an inflammatory biomarker, and hemoglobin level at the end line. Anthropometric parameters were measured according to the WHO growth standards through ENA-SMART software version 2020 [26]. Windiet^®^ (2005) software (The Robert Gordon University, Aberdeen, Scotland, UK) was used to analyze the 24 h dietary recall data for the total energy, macronutrient, and micronutrient composition of all the meals consumed. Multiple linear regression models were used to evaluate the effect of micronutrient powder (MNP) on each of the plasma micronutrients, including plasma zinc, plasma vitamin A, vitamin D, and hemoglobin (Hb) level. The unadjusted model included age and study arm, whereas the adjusted model additionally included child age at enrollment, gender of the child, HAZ, WHZ, WAZ score, plasma albumin status, reported diarrhea in the previous month, reported acute respiratory disease in the previous month, socioeconomic status of households (HHs), breastfeeding in the first hour when a child was born, exclusive breastfeeding, complementary feeding age in months, mother’s age (y), mother’s education, mother’s work status, father’s education, father’s work status, energy consumed (Kcal), and baseline values of each desired outcome variable.

### 2.9. Ethical Consideration

The ethical review board of Khyber Medical University approved the project. Each possible participant was informed about the study’s purpose and components. After gaining informed consent from their parents/guardians, selected children were recruited for the study. The trial was registered under the ISRCTN94319790 number.

## 3. Results

One hundred and fifty households were considered for assessment, among which one hundred and one households were enrolled to participate in the study. Among the 38 children excluded, 31 were excluded due to not meeting the inclusion criteria. Due to concerns about blood collection and families migrating to different locations, eight individuals chose not to participate. The CONSORT diagram is shown in Figure 1.

The baseline characteristics of households, caregivers, and children are shown in Table 2. The mean age of children at baseline was (37.2 ± 4.3 months) in the intervention arm and (36.6 ± 5.6 months) in the control arm. Gender distribution was comparable in both groups. Father education was higher in the control arm compared to the intervention arm. No major differences were observed in the child-feeding practices between the intervention and control arms. There were no noticeable differences between the arms of the study in any other demographic and anthropometric characteristics (Table 2).

Table 3 shows the impact of MNP on the plasma micronutrient status, hemoglobin (Hb) level, and growth parameter of children 24 to 59 months of age. On the assessment of the micronutrients from the blood samples post-intervention, there was a statistically significant increase in plasma zinc, vitamin A, vitamin D, and hemoglobin level in the intervention arm with respect to the control arm (paired sample *t*-test, <0.01). Within the intervention arm, the mean plasma zinc increased from 47.63 µg/dL (±21.48 SD) to 90.0 µg/dL (±21.18 SD); this was followed by plasma vitamin A, i.e., 17.11 ng/mL (±7.49 SD) to 24.68 ng/mL (±7.12 SD), plasma vitamin D, i.e., 25.71 ng/mL (±7.43 SD) to 35.15 ng/mL (±6.43 SD), and hemoglobin level 11.10 g/dL (±1.65 SD) to 13.0 g/dL (±0.64 SD). These associations were statistically significant (*p*-value < 0.05). The mean WAZ and WHZ scores in the intervention arm increased from (mean ±SD) −1.40 (0.50) to −1.05 (0.0.49), and −1.0 (0.88) to−0.40 (1.01), respectively, and were significantly different to the control arm values (paired sample *t*-test, *p*-value < 0.05). There was no statically significant intervention effect on the height-for-age (HAZ) score in the intervention arm compared to the control arm (*p*-value > 0.05). The mean ± SD values of plasma albumin in the intervention group increased from 3.52 (±0.95) to 4.09 (±0.80) (*p* = 0.002). However, no statistically significant change was observed in the plasma albumin of the control arm (*p* > 0.05). Statistically significant increases in plasma vitamin A, vitamin D, plasma zinc, and hemoglobin levels were observed in the intervention arm compared to the control arm. 

Dietary intake assessment showed that the average nutrient intakes of the enrolled children were lower than the recommended nutrient intakes for 2–5-year-old children according to the Pakistan Dietary Guidelines [27] (Table 4).

Multivariable regression adjusted models showed that the intervention group had significantly higher serum levels of all micronutrients and hemoglobin concentration compared to the control group. The adjusted model for plasma zinc concentration revealed that children in the interventional arm had an increase of 33.42 (µg/dL) (CI 23.80–43.0) in the plasma zinc concentration as compared to the control group (adjusted *p*-value < 0.001). The adjusted model for plasma vitamin D level revealed that children in the intervention arm had 4.79 (ng/mL) higher plasma vitamin D levels (CI 1.63–7.95) and 7.57 (ng/mL) higher plasma vitamin A concentration (CI 5.13–10.02 (adjusted *p*-value < 0.05). Similarly, the impact of MNP in the interventional arm showed an increase of 2.0 (g/dL) in hemoglobin level (CI 1.64–2.40) as compared to the control group (adjusted *p*-value < 0.001) (Table 5).

Further analysis showed that the intervention of the micronutrient powder resulted in a 6.8% and 10.35% reduction in wasting and being underweight, respectively, in the intervention group, while there was no significant change (2.01%) in the stunting of children aged 24–59 months. Plasma zinc, vitamin A, vitamin D, and anemia prevalence decreased from 74.07 to 14.04%, 70.18 to 16.36%, 77.19 to 19.30%, and 43.10 to 17.24%, respectively, in the intervention group (Table 6).

## 4. Discussion

This trial showed that one-year supplementation of MNP to 24–59-month-old children significantly improves plasma micronutrient status, hemoglobin levels, and growth parameters, including WAZ and WHZ scores. This is the first community-based study which shows the positive impact of MNP on improving plasma micronutrient levels in a region that has experienced protracted security threats, mass internal population displacement, poor food security, and limited access to quality health services.

Significant improvements in the micronutrient status, growth parameters (WAZ and WHZ scores), and hemoglobin levels of children in this trial are in agreement with findings from other studies [28,29,30]. This study found that MNP supplementation improved the mean vitamin A status among the 24–59-month-old children and reduced the incidence of vitamin A deficiency by 53.82% in the intervention group. Similar to our findings, Suchdev et al. reported a reduction of 7.5% in vitamin A deficiency with MNP supplementation in children aged 6–35 months for 12 months [17]. Chen et al. also studied the impact of multiple micronutrient supplements on 2- to 6-year-old pre-school children for 6 months and found an increase in the vitamin A level from 28.60 to 34.77 ng/mL. This increase in values was similar to our findings, i.e., 17.11 to 24.68 ng/mL for vitamin A with MNP supplementation [29]. In contrast to our findings, Untoro et al. observed no effect of daily MNP supplementation for 23 weeks in children aged 6 to 12 months [31]. Similarly, Ford et al. did not find any effect of MNP supplementation on vitamin A plasma levels in 12–23-month-old children, which was attributed to the homeostatic effect of the serum retinol/RBP on vitamin A deficiency [32] One study conducted in India in 36–66-month-old children showed no increase in serum retinol (vitamin A) [33]. One possible explanation for this difference in findings might be due to the prevalence of vitamin A deficiency at baseline [20,34]. The authors revealed that the lack of effectiveness is due to the low prevalence of vitamin A deficiency in children investigated at baseline because of the prior vitamin A supplementation in these areas. Other factors which could be the possible reasons for the difference in results are the age groups, the composition of MNP, duration of the supplementation, dosage and intake regimen, improper packaging, storage, and quality of the MNP pre-mix.

In our investigation, the administration of MNPs, which contain 5 ug of vitamin D, resulted in an increase in plasma vitamin D level from 25.71 (±7.43) to 35.15 (±6.13) (ng/mL). There is limited evidence regarding the impact of MNP on plasma vitamin D levels in children. Nevertheless, supplementation of food fortified with vitamin D has shown an increase in plasma vitamin D levels in young children. Brett et al. reported similar results from a study among 2–8-year-old children after supplementation of vitamin D-fortified food for 12 weeks. The study revealed that vitamin D-fortified food supplements significantly increased vitamin D concentration by 7 nmol/L in the intervention group [35]. In Mongolia, the trial conducted under World vision in the 2000s for the effectiveness of home-based fortification of complementary foods with sprinkles in an integrated nutrition program was evaluated to address rickets and anemia in children between the age of 6 and 59 months. The outcome of this study showed no significant difference in the overall region of the study population [36]. This lack in the effectiveness of MNP supplementation might be due to such reasons as the low concentration of vitamin D and the occurrence of irreversible visible skeletal deformities in 3–5-year-old children.

Irrespective of our study, limited data have been reported on the effectiveness of MNP supplementation on plasma vitamin D levels in children under the age of 5 years. Hence, there is a need to investigate the impact of MNP on plasma vitamin D levels at the community level.

Findings regarding the increase in serum zinc level in this study are consistent with the results of previously conducted MNP trials in under-five children [37,38]. A randomized control trial, conducted in Vietnam, found a considerable reduction (17.3%) in the plasma zinc level in 6–9-year-old children [39]. A study conducted by García-Guerra et al., which examined the impact of MNP supplements on the serum zinc levels of infants aged 6–12 months, administered 6 days per week for 4 months, showed a significant increase in plasma zinc level [40]. Likewise, in a recent trial, zinc was provided to children aged 6–23 months in three different forms (Preventive zinc, Micronutrient powder, and Therapeutic zinc group) for 32–40 weeks. This study found that children who received 10 mg supplemental zinc/day (Micronutrient powder) showed a significant increase in plasma zinc level and a decline in the prevalence of plasma zinc deficiency by 13% from baseline to end line in the intervention group as compared to the control group [41]. Recently, a study was conducted on 9–11-month-old children in Bangladesh and found a significant increase in zinc plasma levels, particularly zinc provided in tablet form, which reduced the prevalence of zinc deficiency by 42.5%. However, the standard MNP provision only reduces the prevalence of zinc deficiency by 7.9%, which is lower than the value measured in our study (60.03%) [42]. Becquey et al. assessed the effect of intermittent preventive zinc supplementation (IPZS), daily preventive zinc supplementation (DPZS), and therapeutic zinc supplementation (TZS) on the plasma zinc levels of infants between the ages of 6 and 30 months. The mean ± SE plasma zinc level was more significantly increased in the DPZS group (3.9 ± 1.3 μg/dL) than in the TZS (−0.5 ± 1.2 μg/dL) and NIC (−1.2 ± 0.9 μg/dL) groups [43]. The possibility of the higher level of plasma zinc in the DPZS group adhered to compliance with supplementation, while no significant change was observed in the plasma zinc level of the IPSZ and TZS groups, which was attributed to the short-lived effect of change while collecting the blood sample. As compared to these studies, we observed a higher percent reduction of zinc deficiency in the intervention group. These differences could be due to differences in age, duration of supplementation, and composition of the supplements. These findings reveal that MNP supplementation is an effective strategy to combat the silent hunger of micronutrient deficiencies. Nevertheless, other strategies also exist, e.g., fortification/bio-fortification, which have their strengths and advantages in a specific context [44,45].

MNP fortification is one of the public health strategies used around the world to reduce iron deficiency and anemia in children [46,47]. Our clinical trial demonstrated that MNP supplementation was effective in improving the mean hemoglobin levels by (2.0 g/dL) and also significantly reducing anemia prevalence by 29.93%. Our findings are consistent with those of research conducted in Brazil and Mongolia on children aged 6 to 42 months and 6–59 months, respectively [36,48]. Similarly, Kejo et al. studied the impact of micronutrient powder on hemoglobin concentration in children aged 6–59 months in Tanzania. This study found a significant increase in hemoglobin concentration from 9.0 ± 0.70 g/dL at baseline to 11.32 ± 0.52 g/dL at the end line of the study in the children consuming five sachets per week compared to the control group [49]. This finding is similar to the trend found in our results. De-Regil et al. provide a comprehensive review that highlighted the lower risk prevalence of anemia with MNP supplementation in 24–59-month-old children [50]. M Black et al. investigated the effect of point-of-use multiple micronutrient powder (MNP) in 29–49-month-old children and found that meal fortification with MNP reduced the prevalence of anemia and iron deficiency [51]. A study conducted in Nepal by Locks et al. found that areas with high MNP coverage such as Achham district showed a significant reduction in the prevalence of anemia [52]. Contrary to our findings, Oliveira et al. did not find an increase in hemoglobin concentration with MNP supplementation in young Amazonian children, although the iron deficiency was reduced compared to the control [53].This difference in findings with respect to our study might stem from variation in the age of the children, the composition of the supplements, the baseline nutritional status of the target population, the period of the intervention, and other factors responsible for anemia [28].

In this clinical research, stunting (2.01%) in children between the ages of 24–59 months did not significantly improve, although there was a reduction in wasting and underweight, estimated at 6.8% and 10.35%, respectively. A cross-sectional study conducted in Nepal highlighted similar findings with a significant reduction in both wasting and underweight, but not in stunting, after supplementation of MNP [52]. Similar to our findings, except for stunting, Albelbeisi et al. indicated a significant improvement in growth parameters by providing MNP to 6–24-month-old children for 12 months [54]. In our findings, no significant difference in stunting was identified as compared to wasting and underweight. Contrary to our findings, a Cluster Randomized Control Trial conducted on providing MNP supplementation to children aged 24 to 59 months for 6 months in the Thatta and Sujawal districts of the Sindh province was found to have no significant impact on the rates of wasting and underweight. This difference in findings with our results might be due to the period of supplementation [19], age groups, the fact that stunting is normally reversible up to the age of 2 years [54,55], and the occurrence of various diseases such as malaria and diarrhea, and the existence of other forms of undernutrition [56].

We also observed an improvement in plasma albumin levels in the intervention group, while in the control group, even though this biomarker was of lower concentrations, it remained in the normal ranges. Plasma albumin is an acute phase reactant and is influenced by both acute and chronic inflammation which are frequently present alongside micronutrient deficiencies [57]. In our trial, the normal concentration of albumin shows that the assessed levels of the plasma micronutrients reflect the actual nutritional status rather than an altered response to inflammation [58].

Findings from this trial are the first evidence regarding the impact of MNP on multiple micronutrient status including vitamin D, which was not reported earlier.

The macro- and micronutrient intake in 24 h recall data were measured as lower than the recommended dietary intake in light of the Pakistan Dietary Guideline for Better Nutrition recommendations [27]. Given the deprivation and low socioeconomic conditions of the study area, it is difficult to provide children with the necessary food for their healthy development. Determining the usual dietary intake in the community was the goal rather than looking into the potential effects of diet.

This one-year follow-up study evaluated the impact of locally produced MNP nutritional supplements on plasma micronutrient levels, hemoglobin concentration, and growth indicators of children between the ages of 24 and 59 months at the community level. This is the first study in the erstwhile Federally Administrative Tribal Area (FATA) that assesses the impact of MNP on plasma micronutrient status at the community level.

## 5. Conclusions

Locally produced micronutrient powder (local name: Vita-Mixe) supplementation is an effective intervention to increase the micronutrient level, especially plasma zinc, plasma vitamin A, and vitamin D in children. The MNP supplementation significantly improved the WHZ and WAZ scores, while no such statistically significant effect was observed on HAZ scores. It was also effective in improving hemoglobin levels, which is one of the key biomarkers for the child’s nutritional anemia. Micronutrient powder supplementation is, therefore, a cost-effective intervention that can be scaled up among the most food-insecure areas/households to address the alarming rates of undernutrition in Pakistan and other developing countries.

### Strengths and Limitations

The strength of this study is that it is a community-based clinical trial that was successfully completed. There was an uninterrupted supply of MNP to the study participants. The intervention consisted of locally produced nutritional supplements that met the required international standard for MNP. No issue in compliance was reported, resulting in no loss to follow-up over the study course. We also assessed inflammation status among the study participants to ensure that the assessed levels of the micronutrients reflect the actual nutritional status rather than an altered response to inflammation. Limitations of this study include the following: The gold standard in interventional research, random allocation, was not adopted. Nonetheless, distinct regions of the communities were used to prevent contamination between the intervention and control arms of the trial. Sharing of supplements and food is a common practice in local communities; however, MNP intake was closely monitored by Lady Health Workers (LHW), which ensured that the supplements were consumed by the study participant. Dietary assessment was conducted using a single 24 h dietary recall, which may not reflect the usual micronutrient intake of the children. However, the intent was to evaluate average group-level intake. According to the WHO recommendations for assessing the impact of MNP on micronutrient status, it is best to assess the Fe and anemia of the study population, but due to budget limitations, we only assessed anemia. Growth was only measured at two time points; ideally, it would be measured over a period of 3 months to enable consistent changes to be seen. However, due to a lack of resources and sample collection, we were unable to collect growth data over a period of three months.

## Figures and Tables

**Table 1:** Composition Multiple Micronutrient Powder (MNP).

Nutrients Values Per One Gram Sachet	Unit/Day	Quantity	Chemical Form	Recommended Daily Allowances for the Pakistani Population for 24 to 59 Month-Old Children
Retinol (VitaminA)	μg	400	(as dry CWS vitamin A acetate or palmitate beadlets)	300–400
Thiamin (VitaminB1)	mg	0.5	(as Thiamine mononitrate)	0.5–0.6
Riboflavin (Vitamin B2)	mg	0.5	(Riboflavin or riboflavin -5-phosphate)	0.5–0.6
Niacin (VitaminB3)	mg	6	(as Nicotinamide)	6–8
Pyridoxine (Vitamin B6)	mg	0.5	(as Pyridoxine hydrochloride)	0.5–0.6
Folate (VitaminB9)	μg	90	(anhydrous)	150–200
Cobalamin (Vitamin B12)	μg	0.9	(1% or 0.1% Cyanocobalamin on a carrier)	0.9–1.2
Ascorbate (VitaminC)	mg	30	(as sodium or calcium ascorbate)	15–25
Cholecalciferol (Vitamin D)	μg	5	(as dry CWS Cholecalciferol)	8.3–10
Tocopherol Acetate (Vitamin E)	mg aTE	5	(as CWS d or dl-alpha tocopheryl acetate)	6–7
Copper (Cu)	mg	0.7	(as Copper gluconate or sulphate)	340–440
Iodine (I)	μg	90	(as Potassium iodide, or potassium iodate)	90
Iron (Fe)	mg	10	(as coated Ferrous fumerate, NaFe EDTA*or Ferrous bisglycinate)	20
Selenium (Se)	μg	17	(as Sodium selenate or selenite or selenomethionine)	20–30
Zinc (Zn)	mg	4.1	mg (as Zinc sulphate, or gluconate)	15

**Table 2:** Baseline characteristics of households, caregivers, and children by study arm.

Baseline Characteristics	Intervention (n = 58)	Control (n = 53)
Socioeconomic status of * households n (%)		
Poor	31 (53.45)	31 (58.49)
Non-poor	27 (46.55)	22 (41.51)
Mother Age (y) (mean ± SD)	30.21 (5.20)	30.93 (6.45)
Mother Education n (%)		
Formal education > 3 y	14 (24.14)	14 (26.42)
Informal education < 3 y	44 (75.86)	39 (73.58)
Mother Work Status n (%)		
Working woman	6 (10.34)	5 (9.43)
Housewife	52 (89.66)	48 (90.57)
Father Education n (%)		
Formal education	37 (63.79)	39 (73.58)
Informal education	21 (36.21)	14 (26.42)
Father Work Status		
Paid work	42 (72.41)	38 (71.70)
Unemployed	16 (27.59)	15 (28.30)
Family structure n (%)		
Single	21 (36.21)	16 (30.19)
Joint	37 (63.79)	37 (69.81)
Age of child in months	37.2 (4.30)	36.6 (5.60)
Gender of child, n (%)		
Male	28 (48.30)	27 (51.0)
Female	30 (51.7)	26 (49.0)
Childhood specific morbidities (in the previous month)		
Diarrhea n (%)		
No	52 (89.66)	48 (90.57)
Yes	6 (10.34)	5 (9.43)
Acute respiratory infections n (%)		
No	48 (82.76)	45 (84.91)
Yes	10 (17.24)	8 (15.09)
Vaccination status of the child n (%)		
Fully Vaccinated	46 (79.31)	43 (81.13)
Incomplete vaccination	12 (20.69)	10 (18.87)
Breastfeeding in the first hour after birth n (%)		
No	19 (32.76)	14 (26.42)
Yes	39 (67.24)	39 (73.58)
Exclusive Breastfeeding n (%)		
No	8 (13.79)	6 (11.32)
Yes	50 (86.21)	47 (88.68)
Age of complementary feeding introduction (mean ± SD)	7.28 (3.41)	6.85 (2.27)

* A household is a group of persons who make common provisions of food, shelter, and other essentials for living.

**Table 3:** Comparison of plasma micronutrient status, anthropometric outcomes, hemoglobin level, and inflammatory biomarker of children at the baseline and end line by study groups.

Characteristics	Control Baseline (N = 53)	Control End line (N = 53)	Mean Difference	*p*-Value	Intervention Baseline (N = 58)	Intervention End Line (N = 58)	Mean Difference	*p*-Value	DID	*p*-Value
	Mean (SD)	Mean (SD)			Mean (SD)	Mean (SD)				
Growth indicators										
HAZ	−0.94 (1.44)	−1.12 (1.46)	−0.18	0.47	−1.29 (0.88)	−1.30 (0.86)	−0.01	0.93	0.17	0.51
WHZ	−1.32 (1.21)	−1.31 (1.08)	−0.004	0.94	−1.0 (0.88)	−0.40 (1.01)	0.60	<0.001	0.60	<0.001
WAZ	−1.37 (1.0)	−1.51 (0.98)	−0.14	0.35	−1.40 (0.50)	−1.05 (0.49)	0.34	<0.001	0.50	<0.001
Micronutrients status										
Plasma zinc (µg/dL)	49.46 (16.82)	54.17 (15.53)	4.71	0.07	47.63 (21.48)	90.0 (21.18)	42.0	<0.001	37.36	<0.001
Plasma vitamin A (ng/mL)	18.29 (5.57)	18.82 (5.05)	0.52	0.43	17.11 (7.49)	24.68 (7.12)	7.5	<0.001	7.03	<0.001
Plasma vitamin D (ng/mL)	28.20 (10.74)	29.52 (7.41)	1.32	0.33	25.71 (7.43)	35.15 (6.43)	9.44	<0.001	8.12	<0.001
Anemia status										
Anemia Hb (g/dL)	10.7 (1.65)	11.0 (0.94)	0.18	0.50	11.10 (1.65)	13.0 (0.64)	1.84	<0.001	1.67	<0.001
Inflammatory marker										
Serum albumin (g/dL)	3.57 (0.85)	3.41 (0.64)	−0.16	0.30	3.52 (0.95)	4.09 (0.80)	0.56	0.002	0.73	0.002

**Table 4:** Daily dietary nutrient intake during the trial.

Variable	Control Baseline (N = 53)	Control End Line (N = 53)	*p*-Value	Intervention Baseline (N = 58)	Intervention End Line (N = 58)	*p*-Value	Daily Dietary Recommendation PK Guidelines
	Mean (SD)	Mean (SD)		Mean (SD)	Mean (SD)		
Energy (kcal/d)	1144.83 (90.46)	1170.50 (85.08)	0.11	1141.75 (86.70)	1173.05 (99.85)	0.06	1510
Fat (g/d)	28.49 (4.39)	29.00 (4.57)	0.61	27.8 (3.10)	28.75 (4.66)	0.21	30–35
Carbohydrate (g/d)	167.54 (35.17)	167.20 (33.27)	0.93	165.29 (16.5)	163.55 (12.96)	0.49	
Protein (g/d)	22.55 (4.07)	23.16 (3.56)	0.39	22.97 (2.79)	24.22 (4.68)	0.10	26
Vitamin A (µg/d)	190.79 (81.19)	220.75 (86.64)	0.08	213.84 (88.03)	220.93 (86.69)	0.66	400
Vitamin D (µg/d)	0.55 (0.63)	0.56 (0.65)	0.89	0.64 (0.63)	0.73 (0.60)	0.40	8.3
Iron (mg/d)	5.79 (2.20)	6.48 (1.70)	0.09	6.40 (1.93)	6.36 (1.74)	0.85	20
Zinc (mg/d)	5.83 (0.81)	5.62 (0.94)	0.19	5.19 (0.94)	5.35 (1.13)	0.42	15

**Table 5:** Multiple linear regression analysis of the plasma zinc, vitamin A, vitamin D, and hemoglobin against nutritional outcomes by the group during the course of the study.

Models	Results		
	Coefficient (95% CI)		*p*-Value
**Plasma Zinc at end line Intervention group (µg/dL)**			
Unadjusted	35.8	28.8, 43.0	<0.001
Adjusted *	33.42	23.80, 43.0	<0.001
**Plasma vitamin D at end line Intervention group (ng/mL)**			
Unadjusted	5.70	3.02, 8.35	<0.001
Adjusted **	4.79	1.63, 7.95	0.002
**Plasma vitamin A at end line Intervention group (ng/mL)**			
Unadjusted	5.94	3.59, 8.30	<0.001
Adjusted ***	7.57	5.13, 10.02	<0.001
**Hemoglobin g/dL at end line Intervention group**			
Unadjusted	2.04	1.74, 2.3	<0.001
Adjusted ****	2.0	1.64, 2.40	<0.001

Model * is adjusted for child’s age at enrollment, gender of the child, HAZ, WHZ, and WAZ scores, plasma albumin, reported diarrhea in the previous month, reported acute respiratory disease in the previous month, socioeconomic status of HHs, breastfeeding in the first hour when the child was born, exclusive breastfeeding, complementary feeding age in months, mother’s age (y), mother’s education, mother’s work status, father’s education, father’s work status, vaccination status of the child, family structure, energy consumed (Kcal), and plasma zinc levels at the baseline; model ** is adjusted for child’s age at enrollment, gender of the child, HAZ, WHZ, and WAZ scores, reported diarrhea in the previous month, reported acute respiratory disease in the previous month, socioeconomic status of HHs, breastfeeding in the first hour when the child was born, exclusive breastfeeding, complementary feeding age in months, mother’s age (y), mother’s education, mother’s work status, father’s education, father’s work status, vaccination status of the child, family structure, energy consumed (Kcal), and plasma vitamin D levels at the baseline; model *** is adjusted for child’s age at enrollment, gender of the child, HAZ, WHZ, and WAZ scores, plasma albumin, reported diarrhea in the previous month, reported acute respiratory disease in the previous month, socioeconomic status of HHs, breastfeeding in the first hour when the child was born, exclusive breastfeeding, complementary feeding age in months, mother’s age (y), mother’s education, mother’s work status, father’s education, father’s work status, vaccination status of the child, family structure, energy consumed (Kcal), and plasma vitamin A levels at the baseline; and model **** is adjusted for child’s age at enrollment, gender of the child, HAZ, WHZ, and WAZ scores, reported diarrhea in the previous month, reported acute respiratory disease in the previous month, socioeconomic status of HHs, breastfeeding in the first hour when the child was born, exclusive breastfeeding, complementary feeding age in months, mother’s age (y), mother’s education, mother’s work status, father’s education, father’s work status, vaccination status of the child, family structure, energy consumed (Kcal), and hemoglobin levels at the baseline.

**Table 6:** Impact of MNP intervention on stunting, wasting, being underweight, micronutrient deficiencies, and anemia status in children at the baseline and end line of the trial.

Children’s Characteristics	Control Baseline N = 53	Intervention Baseline N = 58	*p*-Value	Control End Line N = 53	Intervention End Line N = 58	*p*-Value
	n (%)	n (%)		n (%)	n (%)	
Nutritional status						
Stunting (HAZ < −2SD)	16 (30.19)	13 (22.41)	0.35	13 (24.53)	12 (20.70)	0.63
Wasting (WHZ < −2SD)	15 (28.30)	8 (13.79)	0.10	14 (26.42)	4 (7.0)	0.005
Underweight (WAZ < −2SD)	14 (26.42)	9 (15.52)	0.15	16 (30.19)	3 (5.17)	<0.001
Micronutrients status						
Plasma zinc n(%)	42 (79.25)	40 (74.07)	0.52	28 (55.0)	8 (14.04)	<0.001
Plasma vitamin A n(%)	29 (55.77)	40 (70.18)	0.11	19 (40.0)	9 (16.36)	0.008
Plasma vitamin D n(%)	33 (63.46)	44 (77.19)	0.11	26 (52.0)	11 (19.30)	<0.001
Anemia status						
Anemia n(%)	29 (54.72)	25 (43.10)	0.22	25 (47.17)	10 (17.24)	0.001

## Data Availability

The raw data of this study that support the conclusion will be made available by the authors without any reservations as per the standard ethical practices of research.
